# Supplementary material for: Behavioural variability and repeatability in adult zebrafish (Danio rerio) using the novel tank dive test
Source: PLoS One. 2025 Oct 27;20(10):e0335308. doi: 10.1371/journal.pone.0335308 (PMC12558523; doi:10.1371/journal.pone.0335308)
Supplement: Attachment — Submitted filename: Response to Reviewers.docx [file pone.0335308.s004.docx]

Dear Academic Editor and Expert Reviewers,

Thank you for the time and effort you have put into suggesting edits and changes to our manuscript. We have addressed all comments below in **bold.** We believe the manuscript is much improved.

Sincerely,

Johnson and colleagues

PONE-D-25-29898

Behavioural Variability and Repeatability in Adult Zebrafish (Danio rerio) Using the Novel Tank Dive Test

PLOS ONE

Editor comments: Thank you for this interesting manuscript. As an avid user of noldus programs myself, could the lab please clarify the frame rate used for ethovision (and whether this was changed as the fish got older)?

**We used a frame rate of 25 frames per second throughout the experiment and have added this to the methods section.**

Journal Requirements:

When submitting your revision, we need you to address these additional requirements.

1. Please ensure that your manuscript meets PLOS ONE's style requirements, including those for file naming. The PLOS ONE style templates can be found at

<https://journals.plos.org/plosone/s/file?id=wjVg/PLOSOne_formatting_sample_main_body.pdf> and

[https://journals.plos.org/plosone/s/file?id=ba62/PLOSOne_formatting_sample_title_authors_affiliations.pdf](https://track.editorialmanager.com/CL0/https:%2F%2Fjournals.plos.org%2Fplosone%2Fs%2Ffile%3Fid=ba62%2FPLOSOne_formatting_sample_title_authors_affiliations.pdf/1/010f0197e9fa3908-c754d620-6fa8-4698-b61e-2ea8146e732d-000000/ved8KqM2C_KOdgpj25ocX4dCJ6wIpUWtrtoaYlRJe8A=218)

2. We note that the grant information you provided in the ‘Funding Information’ and ‘Financial Disclosure’ sections do not match.

When you resubmit, please ensure that you provide the correct grant numbers for the awards you received for your study in the ‘Funding Information’ section.

***Revised.***

3. Thank you for stating the following financial disclosure:
“This research received funding from the Natural Sciences and Engineering Research Council of Canada (NSERC) Discovery Development grant to T.J.H. (05426).”

Please state what role the funders took in the study. If the funders had no role, please state: "The funders had no role in study design, data collection and analysis, decision to publish, or preparation of the manuscript."
If this statement is not correct you must amend it as needed.
Please include this amended Role of Funder statement in your cover letter; we will change the online submission form on your behalf.

***The suggested statement has been added to the financial disclosure.***

4. Please include captions for your Supporting Information files at the end of your manuscript, and update any in-text citations to match accordingly. Please see our Supporting Information guidelines for more information: <http://journals.plos.org/plosone/s/supporting-information>.

[Note: HTML markup is below. Please do not edit.]

5. Review Comments to the Author

Please use the space provided to explain your answers to the questions above. You may also include additional comments for the author, including concerns about dual publication, research ethics, or publication ethics. (Please upload your review as an attachment if it exceeds 20,000 characters)

Reviewer #1: Johnson and colleagues present a nice study of zebrafish behavior across time. They examine the variability and repetability of zebrafish behavior across three ages (90, 120, and 150 dpf) in the novel tank test. Overall, the manuscript is well written and the results are clear. My comments are mostly minor to improve data presentation, to point out some more recent citations they may want to incorporate, and to clarify some of the statistics.

Minor points

Line 136. I believe luminance is measured as candela per meter squared (not meter cubed) as it refers to a surface.

**Thank you for spotting this typo. It has been corrected (line 163).**

Lines 174 and 180. These methods refer to 'period 1' and 'period 2'. However, in the results section and figure (figure 5) the terms 'early' and 'late' are used. I would keep these consistent throughout the paper to make it easier for the reader to understand what 'early' and 'late' refer to when reading the results

**Thank you for catching this oversight. This has been amended to refer to period 1 and 2 as early and late (line 202).**

Lines 189-190 and figure 5. The author's state that significant differences between groups are when the 95% CrIs do not overlap. However, in figure 5 it appears that several 95% CrIs overlap yet the author's state that there are differences between groups. For example, in figure 5A, the results (lines 257-259) state that there is a difference between males and females. But the 95% CrI's overlap. So this does not appear to be a significant difference. However, I'm not that familiar with Bayesian statistics, and so perhaps I'm misunderstanding something here. If so, I would suggest the author's be a bit clearer about what constitutes a 'significant' or relevant difference in the results section.

**Thank you for this question. The proportion overlap between the 95% CrI of two independent estimates provides a conservative estimate of p-value [39], and thus, effects whose pr < 0.05 are also described as being significantly different. We have added this detail to the methods section (line 227-230).**
Line 331. The author's state that "all n=100 fish were then ranked by time spent...". However, I got the impression earlier in this section (lines 311-314) that only 30 fish were used (10 in each group of HAZ, LAZ, and MAZ). Or was it that only the 'top 10, bottom 10, and middle-most 10' fish were used for the different groups. If so, that wasn't entirely clear from the writing in this section.

**Thank you, this was not worded very well. You are correct. It has been revised to explain that 30 fish were selected from the sample (of N = 91) to form the HAZ, MAZ, and LAZ groups (top 10, bottom 10, and middle-most 10), then their behaviour was analyzed to identify any distinct patterns between groups. “To determine whether distinct high, medium, and low anxiety-level groups could be established based on their zone preference, we performed post-hoc rankings based on the cumulative time each fish (N = 91) spent in the bottom zone of the arena at 150 dpf. The 10 male and female zebrafish who spent the most time in the bottom zone were labelled high anxiety (HAZ), while the 10 zebrafish who spent the least time in the bottom zone were labelled low anxiety (LAZ) and the 10 zebrafish falling in the middle were labelled medium anxiety fish (MAZ)” (line 361-367).**

**Afterwards the entire N = 91 sample was divided into HAZ, MAZ, and LAZ groups to see if similar patterns of behaviour were present. However, this was not necessary and seemed to cause confusion, so we have decided to remove this section and associated figures.**

It is not clear when different post-hoc tests are used in different figures/results. In the methods section its stated that sometimes a Dunnet (line 156) or Tukey HSD (line 157) or Fisher's LSD (line 165) is used as a post-hoc test. However, it's not clear in the results when the different tests are used. This is important because, for example, the Fisher's LSD does not manage family wise error rates like the Dunnett or Tukey does. This will help readers better interpret which effects are likely to be more robust.

**The statistical analysis section has been revised to better clarify which analyses were done for each results section.

Line 180-196: “For each behaviour variable, data distributions were assessed using the D’Agostino-Pearson omnibus normality test. Because repeated measures across time within subjects violated normality assumptions, age-related differences were evaluated using the non-parametric Friedman test, followed by Dunn’s multiple comparisons test to identify significant pairwise differences across timepoints. To assess sex differences and age × sex interactions, a two-way ANOVA was conducted for each behavioural measure, treating age and sex as between-subjects factors. Post hoc comparisons were performed using Fisher’s Least Significant Difference (LSD) test. These analyses did not account for within-subject matching and were treated as independent group comparisons across age and sex. Although the data were non-normally distributed, ANOVAs were applied due to their robustness to moderate deviations from normality in balanced designs with large sample sizes. To assess potential effects of repeated exposure, naïve and longitudinal groups were compared at each developmental stage (90, 120, 150 dpf). Because Bartlett’s test indicated unequal variances, Brown–Forsythe ANOVAs were used for these comparisons, followed by Dunnett’s T3 post hoc test. An alpha level of p < .05 and 95% confidence intervals were used to assess statistical significance. All values are presented as mean ± standard error of the mean (S.E.M.).”**

Discussion
Line 353-355. The increase in velocity could also be due to an increase in size. Especially if fish are being housed at the low density indicated here, they are likely to grow quite a bit more between 90 and 120 dpf if they were housed at higher density beforehand. This latter point should also be made.

**Thank you for highlighting how changes in housing density may influence swimming velocity via body size, and for prompting consideration of appropriate references. Recent studies are now cited to support this, and environmental context is emphasized as important in interpreting velocity data. To address this, the Discussion has been amended to read; “Changes in housing density may also contribute to variation in swimming behaviour through effects on body size, as reduced density typically promotes growth. While increased body size is often associated with greater swimming velocity, studies show this relationship is not linear and may depend on morphology, sex, and physiological factors. Furthermore, both housing density and tank size can modulate swimming behaviour, underscoring the importance of considering environmental context when interpreting locomotor data [41-43]. In the present study, only males showed increased swimming velocity across time, whereas females’ velocities remained stable, suggesting that sex-specific differences and environmental effects combine to influence swimming performance” (lines 421-430).**

**Shishis, S., Tsang, B., & Gerlai, R. (2022). The effect of fish density and tank size on the behavior of adult zebrafish: A systematic analysis. *Frontiers in Behavioral Neuroscience*, *16*. https://doi.org/10.3389/fnbeh.2022.934809**

**Palstra, A. P., Tudorache, C., Rovira, M., Brittijn, S. A., Burgerhout, E., van den Thillart, G. E., Spaink, H. P., & Planas, J. V. (2010). Establishing zebrafish as a novel exercise model: Swimming Economy, swimming-enhanced growth and muscle growth marker gene expression. *PLoS ONE*, *5*(12). https://doi.org/10.1371/journal.pone.0014483**

**Miller, C. L., Dugand, R., & McGuigan, K. (2024). Variability of morphology–performance relationships under acute exposure to different temperatures in 3 strains of zebrafish. *Current Zoology*, *71*(2), 152–161. https://doi.org/10.1093/cz/zoae032**

Finally, I would also kindly point the author's to papers we have recently published that touches on several of the topics discussed in this paper. For example, in Rajput et al, 2022 we explicitly study the consistency of individual differences over time and look at strain and sex differences. We show, for example, that males have higher locomotor activity than females (line 368) and that individual differences in behavior are largely consistent over days and weeks (lines 80, 85, and 92). In another paper (Beigloo et al, 2024), we also show that behavioral type can affect behavioral responses to anxiolytic compounds (escitalopram), an idea suggested in the discussion (lines 414-417). That being said, because these papers are from my own lab, it is completely at the discretion of the author and editor whether it is appropriate to include these citations.

References

Rajput, N., Parikh, K., & Kenney, J. W. (2022). Beyond bold versus shy: Zebrafish exploratory behavior falls into several behavioral clusters and is influenced by strain and sex. Biology Open, 11(8), bio059443.

Beigloo, F., Davidson, C. J., Gjonaj, J., Perrine, S. A., & Kenney, J. W. (2024). Individual differences in the boldness of female zebrafish are associated with alterations in serotonin function. Journal of Experimental Biology, 227(12).

**Thank you for directing us to your recent publications. We read both Rajput et al. (2022) and Beigloo et al. (2024) with interest and found them highly relevant to our discussion of individual behavioural consistency, sex differences, and the influence of behavioural phenotype on pharmacological responsiveness. We felt these studies contributed to our interpretation of the results, particularly with regard to trait stability across time and the potential modulatory role of personality traits in drug effects. We have now cited both papers in the revised manuscript and integrated their findings into the discussion.**

**On line 439, we added the following: “These findings align with previous studies which report that male zebrafish generally display greater locomotor activity levels than females [25, 44], possibly reflecting differences in stress coping styles [56].**

**On line 456: “This pattern indicates that anxiety-related and locomotor traits become more stable and trait-like as zebrafish mature, supporting previous findings that behavioural repeatability increases with age [29]. These results are consistent with Rajput et al. [44], who reported that exploratory behaviour in zebrafish remains stable over time and is shaped by both strain and sex.”

On line 475: “Similarly, Beigloo et al. [51] demonstrated that bold and shy female zebrafish exhibit opposing behavioural responses to an anxiolytic compound, highlighting how intrinsic personality traits can meaningfully modulate pharmacological effects, and how variability in novelty response is influenced by sex and developmental stage. Together, these findings suggest that anxiety phenotype may critically influence drug responsiveness and should be considered in the interpretation of pharmacological studies using zebrafish.”**

**On line 490: “These findings align with previous research that demonstrate individual differences in zebrafish behaviours are maintained across biologically meaningful timescales [44] and testing contexts [31].”**

Reviewer #2: This manuscript presents a longitudinal behavioral analysis of adult zebrafish using the Novel Tank Dive Test (NTT), highlighting individual variability and repeatability across development. The study is methodologically ambitious and holds promise to inform future work on zebrafish anxiety-like behavior. However, there are several major concerns regarding the experimental design, interpretation of results, and reproducibility that must be addressed before publication.

Point 1. Habituation and Repeated Testing Effects is a major concern. Although the authors acknowledge the possibility that behavioral changes over time may result from re-exposure rather than developmental progression, they nonetheless interpret their findings as age-related decreases in anxiety-like behavior. This is methodologically problematic.
Repeated testing in the NTT is well known to produce habituation effects, which can mimic anxiolysis. Therefore, it is inappropriate to infer developmental reductions in anxiety without a control group of naïve animals tested only once at each time point. I strongly recommend the authors to either include additional experiments with naïve age-matched groups or substantially reframe their claims.

**We thank the reviewer for raising the important issue of habituation and repeated testing effects in the Novel Tank Dive Test (NTT). We fully acknowledge that in some tests with short intervals between data collection, repeated exposures can induce habituation, potentially mimicking anxiolytic effects. To address this concern and further support our interpretation, we note that our testing intervals were substantial (approximately one month between sessions) rather than massed or frequent exposures. The available literature on larval zebrafish demonstrates that the persistence of habituation is strongly dependent on the interval between exposures: studies have shown that habituation to aversive or novelty-based stimuli in zebrafish larvae decays or even reverses with long intertrial intervals. For example, Xu et al. (2021) found that while a brief (2-hour) gap between dark avoidance trials resulted in typical habituation, a prolonged interval (~22 hours, overnight) led to renewed avoidance, effectively a loss of habituation. Similarly, Beppi et al. (2021) reported that extended delays between startle re-exposures resulted in recovery from habituation in zebrafish larvae. Thus, with sufficiently long intervals between tests, habituation effects can be minimized or extinguished. Although these specific findings have been more thoroughly characterized in larvae, they suggest that the risk of persistent habituation confounding behaviour may be substantially reduced by spacing testing sessions by several days or weeks, as in our study. To further address this issue, we collected additional data from naïve fish tested only once at either 90, 120, or 150 dpf, and also incorporated previously collected data from our laboratory on naïve fish tested in the same manner. Brown–Forsythe ANOVAs revealed no significant differences in time spent in the lower zone between naïve and non-naïve groups at any age (Fig. 2C), indicating that repeated testing did not confound age-related behavioural trends. This analysis is included in the Methods, Results, and Discussion sections of the paper, and the combined naïve cohort data are provided in the supplementary materials.

Line 140: “In addition to the longitudinal cohort, a separate naïve cohort of AB/wild-type hybrid zebrafish was used to control for potential effects of repeated testing. These fish were drawn from the same breeding facility and maintained under the same conditions as described above, but were tested only once at either 90, 120, or 150 dpf. The naïve cohort consisted of n = 20 fish per age group (balanced for sex), and they did not undergo any behavioural testing prior to their single Novel Tank Dive Test exposure.”

Line 191: “To assess potential effects of repeated exposure, naïve and non-naïve groups were compared at each developmental stage (90, 120, 150 dpf). Because Bartlett’s test indicated unequal variances, Brown–Forsythe ANOVAs were used for these comparisons, followed by Dunnett’s T3 post hoc test.”

Line 257: “To evaluate whether repeated exposure influenced behaviour, a naïve cohort of zebrafish tested only once at 90, 120, or 150 dpf was compared to the longitudinally tested cohort. A Brown–Forsythe ANOVA indicated a significant overall effect of group (F(5, 120.6) = 3.92, p = 0.0025), but Dunnett’s T3 post hoc tests revealed no significant differences in time spent in the lower zone between naïve and non-naïve fish at any age (all p > 0.99; Fig. 2C). This suggests that repeated testing did not confound age-related behavioural trends.”

Line 399: “Comparisons with a separate naïve cohort tested only once at each age confirmed that repeated exposure did not drive the observed age-related shifts in vertical exploration. Naïve and longitudinal cohorts did not differ in lower-zone time at any age, indicating that the developmental trends reported here are unlikely to reflect habituation to the test.”**

**Beppi, C., Straumann, D., & Bögli, S. Y. (2021). A model-based quantification of startle reflex habituation in larval zebrafish. *Scientific Reports, 11*(1). https://doi.org/10.1038/s41598-020-79923-6**

**Xu, J., Casanave, R., & Guo, S. (2021). Larval zebrafish display dynamic learning of aversive stimuli in a constant visual surrounding. *Learning &amp; Memory*, *28*(7), 228–238. https://doi.org/10.1101/lm.053425.121**

Point 2. Lack of Arena Randomization and Positional Control is also a problem. There is no description of how the arenas were assigned or randomized across testing days or fish. Since four fish were tested simultaneously, arena position or time-of-day effects could bias results or artificially inflate repeatability. Please, clarify whether randomization procedures were implemented.

**Thank you for this observation. We agree that positional and temporal confounds can affect behavioural outcomes and we did take steps to minimize these effects in our study, which we realize should have been better described. Fish were tested in four identical arenas, with four individuals tested simultaneously per trial. To avoid systematic bias from arena location or time of day, the testing order was rotated across the three developmental timepoints. This ensured that each fish was tested at different times of day and in different arena positions across sessions. This has been added to the Methods section 2.2.**

**Lines 155; “To minimize positional and time-of-day effects, fish were tested in four identical arenas with testing order rotated across developmental timepoints, ensuring that each individual was assessed at different times of day and in different arena positions across sessions.”**

Point 3. The manuscript fails to describe how sex was confirmed. This is especially important in adult zebrafish as external sexual dimorphism can be subtle and unreliable, and gonadal dissection or genetic confirmation is often necessary for accurate assignment. Please clarify how sex was identified, whether through dissection, visual dimorphism, or another method. If not confirmed post-mortem, this introduces potential misclassification bias in sex-based analyses.

**Thank you for raising this point. We initially identified sex based on secondary sexual characteristics at 90 days, including body shape and colouration, which were clearly distinguishable in our adult fish. We validated these characteristics both at 120 and 150 days and saw no discrepancies. Although we did not perform gonadal dissection or genetic confirmation, we noted a consistent pattern during tissue storage: following brain dissection, fish bodies were stored individually in ethanol, and only those identified as male caused the ethanol to turn yellow. While we recognize this method is not validated, the consistency of this pattern provided incidental support for our initial sex assignments. Furthermore, the fish that were later categorized into high-, medium-, and low-anxiety (HAZ, MAZ, LAZ) groups were subsequently used for selective breeding, and all assigned males and females produced viable embryos, further supporting the accuracy of the initial sex classification. We have clarified this point in the revised Methods section 2.1.**

**Line 131: “Sex was identified based on secondary sexual characteristics, including body shape and colouration.”**

Point 4. It is stated that 7 females and 9 males were lost during the study, yet no details are provided regarding the causes, timing, or potential impact on longitudinal behavioral profiles. Given the experimental design, if fish were sharing a tank 1:1, how did the authors proceed with the fish that shared the tank with the one that died? Could that affect the behavior of the remaining fish?

**Thank you for this important question. During the 21-week period, 2 males and 7 females died, either from natural causes or were euthanized due to deteriorating health. These deaths occurred sporadically across the study period and were not concentrated at specific timepoints. All data from deceased individuals were completely removed from the analysis. To prevent social isolation of the surviving fish, a non-experimental fish of the same sex was introduced into the adjoining compartment. These replacements were not included in any analysis. This has been included in the Methods section 2.1.

Line 132: “Throughout the 21-week study, 2 males and 7 females died, either due to natural causes (e.g., senescence, egg binding) or were humanely euthanized following signs of poor health. Mortality occurred sporadically and not at specific timepoints. All data from deceased fish were completely removed from the analysis. To prevent social isolation of the remaining fish, a non-experimental fish of the same sex was introduced into the adjacent compartment; these replacements were not included in any analysis. Therefore, the results presented here are based on n = 48 male and n = 43 female zebrafish for a total sample size of N = 91.”**

Point 5. The division of fish into “low,” “medium,” and “high” anxiety groups based on 150 dpf data is methodologically problematic. Using endpoint data for retrospective grouping can lead to circular reasoning and ignores behavioral plasticity or adaptation over time. For instance, individuals classified as "low anxiety" may simply be more habituated or more adaptable, not inherently less anxious.

**Thank you for raising this important point.** **We agree that grouping individuals based on endpoint behaviour (150 dpf) can raise concerns about circular reasoning, and we appreciate the caution that such an approach may conflate stable trait differences with behavioural plasticity or differential habituation. In our case, the post hoc grouping was intended not to define trait anxiety prospectively, but to identify behavioural profiles that had emerged by the final timepoint, with the broader goal of informing future selective breeding efforts. After assigning individuals to high-, medium-, and low-anxiety groups based on lower-zone time at 150 dpf, we examined how those same individuals behaved at 90 and 120 dpf with the goal of examining whether consistent behavioural patterns could be traced across earlier timepoints. The observation that group-level differences were already evident at earlier timepoints and became more pronounced over time suggests that the behavioural divergence reflects a developmental trajectory, rather than a transient state or difference in habituation rate. However, we acknowledge the limitations of retrospective classification and have clarified the rationale in the discussion.

Line 498: “While the anxiety group classifications were applied retrospectively based on behaviour at 150 dpf, the goal was to explore whether distinguishable behavioural profiles had emerged by late adolescence. This approach does not define trait anxiety prospectively but allows for examination of whether consistent group-level patterns were already present earlier in development. Notably, behavioural differences between groups were evident at 90 and 120 dpf, supporting the interpretation that these groupings captured stable behavioural tendencies rather than short-term fluctuations or habituation effects. Given the exploratory nature of this classification, future studies using prospective grouping or selective breeding designs will be important for validating the developmental stability of these phenotypes.”**

Point 6. Two recent studies that directly relate to the present work are not cited but are essential to contextualize the findings. One study (doi: 10.1242/bio.059443) examined individual exploratory behavior in a novel environment over time, revealing consistent inter-individual differences and reinforcing the importance of accounting for stable behavioral phenotypes in repeated-measures designs. Another study (doi: 10.1371/journal.pone.0300227) found that individual variability in novelty response is influenced by strain, sex, and age, which directly informs the present study’s focus on behavioral repeatability and developmental trajectories. Please revise the reference list to include relevant studies and ensure that other foundational papers on sex differences in zebrafish behavior, particularly in the Novel Tank Test, are cited, as some seminal articles appear to be missing.

**We thank the reviewer for drawing our attention to these important and highly relevant studies. We have now cited Rajput et al. (2022) and Beigloo et al. (2024) in the Discussion section. Rajput et al. is referenced in support of our findings on increasing among-individual variation and behavioural repeatability with age. Beigloo et al. is now used to contextualize our observations of sex- and age-related variation in novelty response, as well as the relevance of stable behavioural traits for interpreting pharmacological sensitivity.**

**On line 439, we added the following: “These findings align with previous studies which report that male zebrafish generally display greater locomotor activity levels than females [25, 44], possibly reflecting differences in stress coping styles [45].**

**On line 456: “This pattern indicates that anxiety-related and locomotor traits become more stable and trait-like as zebrafish mature, supporting previous findings that behavioural repeatability increases with age [29]. These results are consistent with Rajput et al. [44], who reported that exploratory behaviour in zebrafish remains stable over time and is shaped by both strain and sex.”

On line 475: “Similarly, Beigloo et al. [51] demonstrated that bold and shy female zebrafish exhibit opposing behavioural responses to an anxiolytic compound, highlighting how intrinsic personality traits can meaningfully modulate pharmacological effects, and how variability in novelty response is influenced by sex and developmental stage. Together, these findings suggest that anxiety phenotype may critically influence drug responsiveness and should be considered in the interpretation of pharmacological studies using zebrafish.”**

**On line 490: “These findings align with previous research that demonstrate individual differences in zebrafish behaviours are maintained across biologically meaningful timescales [44] and testing contexts [31].”**

**We have also reviewed the literature and added citations to several seminal studies examining sex differences in zebrafish behaviour, including in the Novel Tank Test. These include highly cited works by Genario et al. (2020), Mustafa et al. (2019), and Fontana et al. (2020), which collectively provide strong evidence for sex-dependent differences in anxiety-like and exploratory behaviours in zebrafish.**

**Line 69: “For example, older zebrafish often exhibit reduced baseline locomotion compared to younger fish, and both age and sex differences have been shown to affect anxiety-like and locomotor behaviours [25, 26]. However, sex effects in zebrafish behaviour are not uniform across strains or contexts. For example, Mustafa et al. [27] found that strain identity strongly modulated sex differences in boldness and anxiety-like behaviour.”**

**Line 441: “However, other work has shown that female zebrafish may also exhibit greater activity alongside increased anxiety-like behaviour in the novel tank test [26, 46], highlighting the complexity of sex-specific behavioural profiles.”**

**Genario, R., de Abreu, M. S., Giacomini, A. C. V. V., Demin, K. A., Kolesnikova, T. O., Collier, A. D., et al. (2020). Sex differences in adult zebrafish anxiolytic-like responses to diazepam and melatonin. *Progress in Neuro-Psychopharmacology and Biological Psychiatry, 96*, 109752. doi:10.1016/j.pnpbp.2019.109752**

**Fontana, B. D., Mezzomo, N. J., Kalueff, A. V., & Rosemberg, D. B. (2020). Female adult zebrafish (Danio rerio) show higher levels of anxiety-like behavior than males, but do not differ in learning and memory capacity. *European Journal of Neuroscience, 52*(7), 2635–2645. https://doi.org/10.1111/ejn.14640**

**Mustafa, A., van den Bos, R., & Kloet, R. E. (2019). Boldness in male and female zebrafish (Danio rerio) is dependent on strain and context. *Behavioural Processes, 164*, 169–176. https://doi.org/10.1016/j.beproc.2019.05.011**

Point 7. Anxiety-like behavior in zebrafish, particularly in older individuals, may not follow the same trajectory or behavioral signatures as in larvae or juveniles. The Novel Tank Test (NTT) was originally validated for acute anxiogenic responses, and its use as a developmental or trait-based marker of anxiety in adult zebrafish is still under debate. In this context, it is unclear why the authors chose time spent in tank zones (e.g., upper vs. lower) as the primary behavioral feature to stratify individuals into anxiety-level groups, while other relevant parameters such as immobility, freezing-like behavior, or overall activity were not used as equally weighted indicators.

**We thank the reviewer for raising this important point. While we agree that anxiety-like behaviour is multifaceted and may be expressed through various behavioural parameters, we chose bottom zone time as our primary stratification variable based on its strong empirical support as a stable marker of anxiety-like behaviour in adult zebrafish. In particular, in a past study we (Johnson et al., 2023) conducted a principal component analysis (PCA) on a range of of behavioural measures and found that time spent in the bottom zone loaded most strongly on the first principal component, which explained the majority of behavioural variance and captured core anxiety-like traits. Other measures such as immobility and freezing contributed less prominently to this axis. This suggests that vertical zone preference reflects a central dimension of zebrafish anxiety phenotypes and is an appropriate metric for stratifying individuals. Furthermore, vertical positioning in the novel tank test has consistently been shown to differentiate anxiogenic and anxiolytic states across multiple studies and drug treatments. While freezing and immobility may also reflect fear-like responses, they are often not in line with zone preferences (i.e. Scatterty & Hamilton, 2024), which can also be harder to interpret across developmental stages. Given our focus on consistent trait-like differences across generations, we prioritized a variable with demonstrated reliability, dimensional clarity, and translational relevance.**

**Johnson A, Loh E, Verbitsky R, Slessor J, Franczak BC, Schalomon M, et al. Examining behavioural test sensitivity and locomotor proxies of anxiety-like behaviour in zebrafish. Scientific Reports. 2023;13. doi:10.1038/s41598-023-29668-9

Scatterty, K.R., Hamilton, T.J., 2024. В-carboline (FG-7142) modulates fear but not**

**anxiety-like behaviour in zebrafish. Sci. Rep. 14 (1) https://doi.org/10.1038/**

**s41598-023-51072-6.**

Furthermore, while zone-based scoring is conventional, the use of continuous variables such as distance from bottom may offer a more robust and fine-grained analysis of vertical exploration, without the need to define arbitrary cutoffs or classify subjects post hoc. This would also allow for correlational or trajectory-based analyses of anxiety-like behavior over time, strengthening the claim of trait-level consistency. The authors should justify their prioritization of zone occupancy as the main behavioral classifier, clarify whether immobility or other anxiety-related metrics were considered in combination, and consider re-analyzing or presenting vertical position as a continuous measure rather than a categorical one. This may offer greater sensitivity to developmental or individual differences and avoid potential oversimplification of complex behavioral traits.

**We based our analysis of vertical exploration on the conventional method of defined zone regions, as time spent in the lower zone is a widely validated measure of anxiety-like behaviour in zebrafish. Previous work, including Johnson et al. (2023), has shown through principal component analysis (PCA) that lower-zone time loads most strongly on the main axis of anxiety-like traits, supporting its use as a primary classifier. The continuous variable *distance from bottom* was also quantified by extracting the mean vertical position of each fish across the 10-minute trial. A Friedman test indicated a significant effect of age (H(2) = 14.0, p = 0.0009), consistent with the zone-based analysis, but this measure did not provide more robust statistical differences between groups. In this case, distance from bottom does not appear to offer greater sensitivity, but we agree that it can be very useful when combined with immobility or other metrics. We have added this clarification to the Results and Discussion.

Line 251: Consistent with these zone-based measures, distance from bottom also showed a significant effect of age (H(2) = 14.0, p = 0.0009), with zebrafish at 150 dpf positioned higher in the water column than at 90 (p = 0.0008) and 120 dpf (p = 0.028), whereas 90 and 120 dpf did not differ (p = 0.898; Fig. 2C). However, distance from bottom did not reveal additional differences beyond those detected with lower zone occupancy.**

**Line 402: "We also analyzed distance from bottom as a continuous measure of vertical exploration. This metric produced results consistent with the zone-based analysis, showing that zebrafish at 150 dpf were positioned higher in the water column compared to 90 and 120 dpf, but it did not reveal additional differences beyond those detected with zone occupancy. This supports the validity of zone occupancy as a robust and interpretable indicator of anxiety-like behaviour, while acknowledging that continuous measures may be useful in combination with other behavioural metrics in future work."**

**Johnson A, Loh E, Verbitsky R, Slessor J, Franczak BC, Schalomon M, et al. Examining behavioural test sensitivity and locomotor proxies of anxiety-like behaviour in zebrafish. Scientific Reports. 2023;13. doi:10.1038/s41598-023-29668-9**

Point 8. The manuscript does not provide a rationale for the chosen sample size (n = 100; 50 males and 50 females). While this is a commendably large N for a behavioral longitudinal study, no information is provided regarding power analysis, effect size expectations, or justification based on previous studies.

**We thank the reviewer for raising this important point. To clarify, our sample size of *n* = 100 (50 males, 50 females) was determined based on feasibility and resource availability, while also exceeding the sample sizes used in comparable zebrafish behavioral studies. To formally assess the adequacy of our sample size, we conducted a power analysis using G*Power (v3.1.9.7; Faul et al., 2007). For the within-subjects (repeated-measures) analyses across three timepoints, assuming a moderate correlation among repeated measures (r = 0.5), α = 0.05, and a medium effect size (f = 0.25), the achieved power was 0.87. This suggests our design was well-powered to detect medium-sized effects over time. For the between-subjects comparisons (i.e., sex differences), we computed power for a two-group (males vs. females) comparison with α = 0.05 and a medium effect size (Cohen’s *d* = 0.5), which yielded a power of 0.80. These values confirm that the sample size was sufficient for the primary aims of this study. We have now added this justification to the Methods section.

Line 231: “A priori power analysis was conducted using G*Power (v3.1.9.7) [40] to evaluate the adequacy of the sample size. For the repeated-measures analyses across three timepoints, assuming a moderate correlation among repeated measures (r = 0.5), α = 0.05, and a medium effect size (f = 0.25), the sample size of n = 100 (50 males, 50 females) yielded a power of 0.87. For between-group comparisons by sex, using α = 0.05 and a medium effect size (Cohen’s d = 0.5), the power was 0.80. These estimates indicate that the study was sufficiently powered to detect medium-sized effects both within and between subjects.”**

**Faul F, Erdfelder E, Lang AG, Buchner A. G*Power 3: A flexible statistical power analysis program for the social, behavioral, and biomedical sciences. *Behav Res Methods*. 2007;39(2):175–191. doi:10.3758/bf03193146**

Reviewer #3: This study assessed individuality and variability across adult zebrafish, with observation spanning across 2 months, using the novel tank dive test. It highlights the need to consider age, sex, and individual variation when conducting zebrafish behavioral assays. Overall, I am very enthusiastic about this manuscript.

Here are my comments:

Were experimental fish housed individually?

**We appreciate the reviewer’s question regarding housing conditions and realize it is unclear in the manuscript. Experimental fish were housed in pairs within 3.5 L tanks that were divided using transparent perforated polycarbonate inserts (one fish on each side). This setup enabled individual housing for behavioural identification without invasive tagging methods or full isolation, while allowing continuous visual and olfactory cues. This minimized social isolation without compromising individual tracking. This has been described better in the methods section and included a reference from Daniel & Bhat, 2022 who demonstrate that visual and olfactory conspecific cues mitigate adverse effects of partial isolation.

Line 120: “To identify individuals during the longitudinal testing period, zebrafish were housed in pairs (1:1 male to female) in 3.5 L polycarbonate tanks separated by transparent, perforated polycarbonate inserts, with each compartment containing plastic plant and floor enrichments (Fig. 1A). While fish were physically separated to enable individual identification across repeated behavioural sessions, the dividers allowed continuous visual and olfactory contact. This housing strategy aligns with evidence that sensory access to conspecifics mitigates the behavioural effects of partial isolation in zebrafish [32].”**

**Daniel DK, Bhat A. Alone but not always lonely: Social cues alleviate isolation induced behavioural stress in wild zebrafish. Applied Animal Behaviour Science. 2022;251: 105623. doi:10.1016/j.applanim.2022.105623**

Just out of curiosity – how did the authors deal with the egg-bound issue of female fish over this 2-month period?

**To mitigate egg-binding, we placed spawning substrates in each tank to facilitate natural egg release. In a few cases where females appeared egg-bound, they were removed from the dataset prior to any intervention. Although manual egg expression was attempted under anesthesia, these procedures were unsuccessful, and the affected individuals were humanely euthanized.**

**Line 124: “To mitigate eggbinding, spawning substrates were placed in each tank to facilitate natural egg release. In a few cases where females appeared egg-bound, they were removed from the dataset prior to any intervention.”**

9 fish were dead, and the data were not included. Thus, the “n” in the results should be reflected accordingly, instead of “n = 50” or “n = 100”.

**Thank you for this important observation. We have revised the manuscript to clarify that 9 fish (2 males, 7 females) died or were euthanized during the course of the study and were excluded from all behavioural analyses. The sample sizes in the manuscript have been updated to reflect the final number of individuals analyzed (n = 48 males, n = 43 females, N = 91 total).

Line 132: “Throughout the 21-week study, 2 males and 7 females died, either due to natural causes (e.g., senescence, egg binding) or were humanely euthanized following signs of poor health. Mortality occurred sporadically and not at specific timepoints. All data from deceased fish were completely removed from the analysis. To prevent social isolation of the remaining fish, a non-experimental fish of the same sex was introduced into the adjacent compartment; these replacements were not included in any behavioural testing or analysis. Therefore, the results presented here are based on n = 48 male and n = 43 female zebrafish for a total sample size of N = 91.”**

**Line 361: “To determine whether distinct high, medium, and low anxiety-level groups could be established based on their zone preference, we performed post-hoc rankings based on the cumulative time each fish (N = 91) spent in the bottom zone of the arena at 150 dpf.”**

Could the author comment on why the locomotion was increased at 90 dpf, compared to 120 dpf, given that the fish were younger and presumably smaller at 90 dpf?

**We thank the reviewer for this question. Locomotion was somewhat higher at 90 dpf compared to 120 dpf, despite fish being smaller at the earlier age. As discussed in our response to Reviewer 1, we have revised the Discussion to clarify how housing density, growth, and body size can influence swimming velocity, and to note that this relationship is not strictly linear but depends on morphology, sex, and environmental factors. These revisions highlight that multiple factors, rather than size alone, likely contribute to the observed velocity differences.

Line 421: “Changes in housing density may also contribute to variation in swimming behaviour through effects on body size, as reduced density typically promotes growth. While increased body size is often associated with greater swimming velocity, studies show this relationship is not linear and may depend on morphology, sex, and physiological factors. Furthermore, both housing density and tank size can modulate swimming behaviour, underscoring the importance of considering environmental context when interpreting locomotor data [41-43]. In the present study, only males showed increased swimming velocity across time, whereas females’ velocities remained stable, suggesting that sex-specific differences and environmental effects combine to influence swimming performance.”**

**Shishis, S., Tsang, B., & Gerlai, R. (2022). The effect of fish density and tank size on the behavior of adult zebrafish: A systematic analysis. *Frontiers in Behavioral Neuroscience*, *16*. https://doi.org/10.3389/fnbeh.2022.934809**

**Palstra, A. P., Tudorache, C., Rovira, M., Brittijn, S. A., Burgerhout, E., van den Thillart, G. E., Spaink, H. P., & Planas, J. V. (2010). Establishing zebrafish as a novel exercise model: Swimming Economy, swimming-enhanced growth and muscle growth marker gene expression. *PLoS ONE*, *5*(12). https://doi.org/10.1371/journal.pone.0014483**

**Miller, C. L., Dugand, R., & McGuigan, K. (2024). Variability of morphology–performance relationships under acute exposure to different temperatures in 3 strains of zebrafish. *Current Zoology*, *71*(2), 152–161. https://doi.org/10.1093/cz/zoae032**

I am not sure if I understand the interpretation of Fig.4. The error bars are mostly overlapping. Are these reported trends (such as “decline”, “reduction”) significant? If not, how are the conclusions justified?

**We thank the reviewer for this observation. The significance of the reported trends is not determined from Figure 4 itself, but from the mixed-effects model outputs that are fully reported in Tables 1–3 and described in the Results section. These tables specify the parameter estimates, 95% credible intervals, and pr values. Figure 4 was included only to illustrate the model-predicted trajectories, but we agree that the overlapping error bars may give the impression that the statistical support for these effects is unclear. To avoid this confusion and reduce redundancy with Figure 3, we have removed Figure 4. All relevant statistical inferences remain reported in the tables and text, and the conclusions are based on these model outputs rather than the figure.**

Fig 5, if variance was measured here, should the unit of the y-axis be “squared”?

**The y-axis do indeed represent variance components. However, variances are in fact unitless. We realize that our labelling of the y-axis may have been confusing, as we report the response variable (and it’s units). To address this comment, we have enriched the descriptions provided in the Figure legend (line 738). Note, this is now Fig 4.**

A couple of comments on statistical analysis:

Line 189-191: I am not sure why would non-overlapping 95% credible interval would correspond to p<0.006 (credible interval is based on Bayesian, p value is based on frequentist). Please elaborate.

**Here, p refers to the proportion of overlap between two 95% CrIs. Because 2.5% of estimates lie outside either side of a 95% CrI, two non-overlapping 95% CrIs can have non-zero overlap of estimates (between 0 and 2.5%). A detailed description and demonstration of inferring p-values from the degree of overlap between confidence (or credible) intervals is provided in Cummings (2008), which we have cited in the manuscript. We have also further clarified on lines 227. To avoid confusion with frequentist p-values, we use ‘pr’ to refer to calculated proportion overlaps between confidence intervals.**

**Cumming G. Inference by eye: Reading the overlap of independent confidence intervals. Statistics in Medicine. 2008;28: 205–220. doi:10.1002/sim.3471**

Could the authors please add more details about what the sim function of the arm package and the MCMCglmm package are and why they are used?

**The sim function from the arm package in R uses flat, uninformative priors to simulate the posterior distribution of estimates from lmer models. This allows us to generate 95% credible intervals (CrIs) of the estimated effects, which can then be used to evaluate the degree of overlap between estimates. The MCMCglmm package was used to obtain 95% CrIs from 1,000 posterior simulations, providing a direct way to assess both the magnitude of effects and the confidence in differences between them. We have added this clarification to the Methods section.**

**Line 209: “We used the sim function of the arm package [33] to simulate values of the posterior distribution of the model parameters [34]. This function applies flat, uninformative priors to lmer estimates, allowing posterior distributions of parameters to be generated and 95% credible intervals (CrIs) to be calculated. These CrIs provide a way to directly evaluate the extent of overlap between estimates, which in turn indicates both whether differences are supported and the magnitude of those differences.”**
